# Association of Diagnosis Age with Clinical Characteristics and MRI Findings in Multiple Sclerosis

**DOI:** 10.3390/neurosci7050111

**Published:** 2026-10-09

**Authors:** Omid Mirmosayyeb, Sepehr Aghajanian, Fateme Mohammadifard, Aram Zabeti, Vahid Shaygannejad

**Affiliations:** 1 Department of Neurology, University of Cincinnati, Cincinnati, OH 45221, USA; Mirmosod@ucmail.uc.edu (O.M.); zabetiam@ucmail.uc.edu (A.Z.); 2 Isfahan Neurosciences Research Center, Isfahan University of Medical Sciences, Isfahan 81839-83434, Iran; s.aghajanian@abzums.ac.ir (S.A.); fa.mohammadiima@gmail.com (F.M.); 3 Department of Neurology, School of Medicine, Isfahan University of Medical Sciences, Isfahan 8174673461, Iran

**Keywords:** multiple sclerosis, demyelinating disease, age at diagnosis, disease association, MRI, aging

## Abstract

Background: Age at multiple sclerosis (MS) diagnosis may be associated with clinical course, inflammatory activity, and disability accrual, but evidence across the full age spectrum remains limited. We compared clinical and radiological features, phenotype distribution, and time to first post-diagnosis relapse across early-onset MS (EOMS), adult-onset MS (AOMS), and late-onset MS (LOMS). Methods: This observational study included patients with MS treated at two tertiary centers in Isfahan, Iran, combining a retrospective review of clinical records with a cross-sectional assessment at the most recent study visit. Patients were categorized as EOMS (MS diagnosis at ≤18 years), AOMS, or LOMS (diagnosis at or after 50 years) according to age at diagnosis. Demographic, clinical, MRI, and comorbidity data were compared across groups. Time to first post-diagnosis relapse was analyzed in RRMS using Cox regression, and continuous age effects were explored with spline models. Results: Among 3994 patients, 257 had EOMS, 3587 had AOMS, and 150 had LOMS. EOMS had a higher hazard of first post-diagnosis relapse (adjusted HR: 1.27, 95% CI: 1.05–1.54) and more frequent enhancing brain lesions. LOMS had a more progressive phenotype, higher EDSS at diagnosis and qualitative brain atrophy, and more age-associated comorbidities. The adjusted LOMS relapse estimate (HR: 0.58, 95% CI: 0.34–0.99) was borderline significant. Progressive MS became more common with increasing diagnosis age, largely reflecting PPMS, although unequal follow-up and last-visit classification limit causal interpretation. Female predominance persisted across all groups but was less pronounced in LOMS. Conclusions: Age at diagnosis was associated with clinical and radiological differences across the MS spectrum from a more relapsing phenotype to a more progressive, disability-associated phenotype. These findings should be verified in prospective studies based on symptomatic onset age.

## 1. Introduction

Multiple sclerosis (MS) is a chronic inflammatory and neurodegenerative disorder of the central nervous system characterized by substantial heterogeneity in clinical presentation, radiological features, and disease course. Although MS most commonly manifests in young adulthood, disease onset can occur from childhood to later life. Age is increasingly recognized as an important determinant of MS phenotype, with previous studies demonstrating age-related differences in presenting symptoms, relapse characteristics, recovery, and the development of progressive disease [1,2,3].

MS occurring before 18 years of age is generally classified as pediatric- or early-onset MS (EOMS), whereas onset at or after 50 years is commonly classified as late-onset MS (LOMS), with cases occurring between these thresholds considered adult-onset MS (AOMS) [4]. LOMS, defined as disease diagnosis at or after the age of 50 years, has a reported prevalence ranging from 0.6% to 12%, accounting for approximately 5% of total MS cases [5,6]. EOMS is generally characterized by a predominantly relapsing and inflammatory disease course, whereas LOMS has been associated with greater disability at presentation and a higher frequency of progressive disease. These differences have prompted increasing interest in the role of age in shaping MS expression. Although age at symptom onset is conventionally used to define these groups, age at diagnosis has also been used as an operational measure in clinical and registry studies [7,8], reflecting differences in the availability of clinical data and inconsistent and unreliable onset symptom definition and timing.

Previous studies, which have compared the clinical characteristics and outcomes of patients with EOMS, AOMS, and LOMS, have established important differences between these age-defined groups. However, categorizing a continuous biological variable such as age into predefined thresholds may obscure changes occurring across the intervening age spectrum. Consequently, group-based comparisons alone cannot establish whether EOMS, AOMS, and LOMS represent sharply distinct clinical phenotypes or points along a broader age-related continuum of changing inflammatory and progressive disease expression. Moreover, relatively few studies have examined clinical phenotype, disability, radiological characteristics, and relapse outcomes together across all three age groups, and such data remain limited in Middle Eastern populations.

Accordingly, the present study aimed to compare the demographic, clinical, radiological, and relapse-related characteristics of patients with EOMS, AOMS, and LOMS in a large MS cohort. In addition to conventional categorical comparisons, age at diagnosis was examined as a continuous variable to determine whether changes in progressive disease phenotype and post-diagnosis relapse risk extended across the age spectrum rather than being confined to predefined onset-group boundaries.

## 2. Materials and Methods

### 2.1. Study Design and Setting

This observational study was conducted between January 2018 and December 2024 at academic tertiary care centers for MS, the Kashani Hospital, and the Hakim MS Clinic in Isfahan, Iran, combining a retrospective longitudinal review of clinical records with a cross-sectional assessment at the most recent study visit. Inclusion criteria were patients diagnosed with MS regardless of age. Patients were diagnosed with MS based on the latest version of the McDonald criteria available at the time of diagnosis [9,10,11]. Cases diagnosed with clinically isolated syndrome (CIS) or radiologically isolated syndrome (RIS), Neuromyelitis Optica Spectrum Disorder, transverse myelitis, or other neurological diagnoses, as well as those with missing diagnosis age, were excluded. The study was performed in accordance with the Declaration of Helsinki and its amendments and was approved by the Ethics Committee of Isfahan University of Medical Sciences (IR.ARI.MUI.REC.1401.061).

### 2.2. Data Collection

Clinical and demographic data were obtained from medical records at diagnosis and at the last follow-up visit. At the last follow-up, patients were interviewed in person, and their medical records were reviewed to collect information on current demographic characteristics, anthropometric measures, comorbidities, MS course, treatment plan, and MRI features. Age at diagnosis was calculated as the interval between date of birth and the year of MS diagnosis. Education was categorized as low (illiterate or those with primary school education), secondary (attaining diploma), and higher (university degrees including bachelor’s and higher). Disease duration was calculated as the interval between the diagnosis year and the date of the last follow-up visit. Relapse outcomes were assessed only in patients with relapsing–remitting MS (RRMS). MS course/subtype was determined at last follow-up. Further details for data acquisition and variable definitions for this work are as previously reported [8]. The present study extends this work by a large sample size, examining time to first post-diagnosis relapse, modeling age at diagnosis continuously, and evaluating adjusted associations with contemporary MRI characteristics across the diagnosis age spectrum.

MRI was performed using a 1.5-T Siemens system and reviewed by a certified neurologist. MRI variables included lesion topography, presence of active plaques, global lesion burden, and visually reported brain atrophy. Atrophy was defined by increased CSF-space prominence and/or reduction in cerebral parenchymal volume. Consistent with previous studies, atrophy was graded using a three-level visual Likert scale—mild (<10%), moderate (10–25%), or severe (>25%)—based on the apparent extent of ventricular enlargement and parenchymal volume reduction [12]. Active brain plaques were defined as gadolinium-enhancing lesions on T1-weighted contrast-enhanced MRI corresponding to plaques visible on other structural 1.5T MRI sequences on the last scan performed before the last follow-up visit. The interval between the MRI scans used for study assessments and follow-up was not allowed to exceed 6 months. Relapse was defined as a new or worsening neurological symptoms diagnosed by the neurologist, lasting at least 24 h, in the absence of fever or infection, and separated from the previous event by at least 30 days. Time to first post-diagnosis relapse was calculated from diagnosis year to the date of the first relapse and was expressed in years. Patients who did not experience a relapse were censored at the last available follow-up. Participants from the study centers were recruited using convenience sampling to facilitate recruitment and obtain the largest feasible sample size, thereby increasing statistical power.

### 2.3. Statistical Analysis

Patients were categorized according to age at clinical diagnosis. EOMS was defined as diagnosis at ≤18 years, AOMS as 18 < diagnosis age < 50 years of age, and LOMS as MS diagnosis age ≥ 50. Baseline characteristics were summarized across the three groups. Continuous variables were reported as mean and standard deviation or median and interquartile range, depending on distribution. Categorical variables were reported as number and percentage. Group comparisons were performed using one-way ANOVA for normally distributed variables and Kruskal–Wallis tests for non-normally distributed variables, while categorical variables were compared using chi-square tests.

Time to first post-diagnosis relapse was analyzed using Cox proportional hazards regression after excluding progressive MS cases. Two categorical diagnosis age-group models were fitted: an unadjusted model and a model adjusted for sex and EDSS at earliest diagnostic visit. While there was a violation of the Cox proportional hazards model for the EOMS term (*p* = 0.0041), the assumption was held for LOMS (*p* = 0.640) and the continuous diagnosis age term (*p* = 0.883). Therefore, the hazard ratio for LOMS can be interpreted under the proportional hazards assumption, whereas the hazard ratio for EOMS should be interpreted cautiously because the relative hazard associated with EOMS varied over follow-up. To account for confounding correlation between MS-related tissue injury and vascular and metabolic comorbidity with brain atrophy, differences between the study groups accounted for the aforementioned factors using an ordinal regression model (with brain atrophy levels as outcome) adjusted for sex, MS subtype, EDSS at earliest diagnostic visit, disease duration, hypertension, and diabetes mellitus. There was no evidence that the proportional odds assumption of the primary model was violated based on the Brant test (*p* = 0.132). The difference in the presence of enhancing brain lesions (active brain plaques) was also further investigated by adjusting for disease duration and MS subtype in logistic regression. Age was not considered as an adjustment factor, as the term collides with the role of age in the definition of the study groups. AOMS was used as the reference group in all regression analyses.

Restricted cubic spline Cox models were used to visualize the association between continuous age at clinical diagnosis and relapse hazard. Four knots were used, and age 30 years was selected as the reference value. Spline analyses were performed in the RRMS subset. Estimated marginal probabilities from adjusted logistic regression were used to model the probability of progressive MS versus RRMS across the age-at-diagnosis spectrum at mean covariate values. Descriptive statistics were calculated using all available non-missing observations for each variable. Regression analyses were conducted using complete-case analysis. All analyses were performed in Stata version 18. Statistical significance was set at a two-sided *p*-value of less than 0.05.

## 3. Results

**Figure 1:**
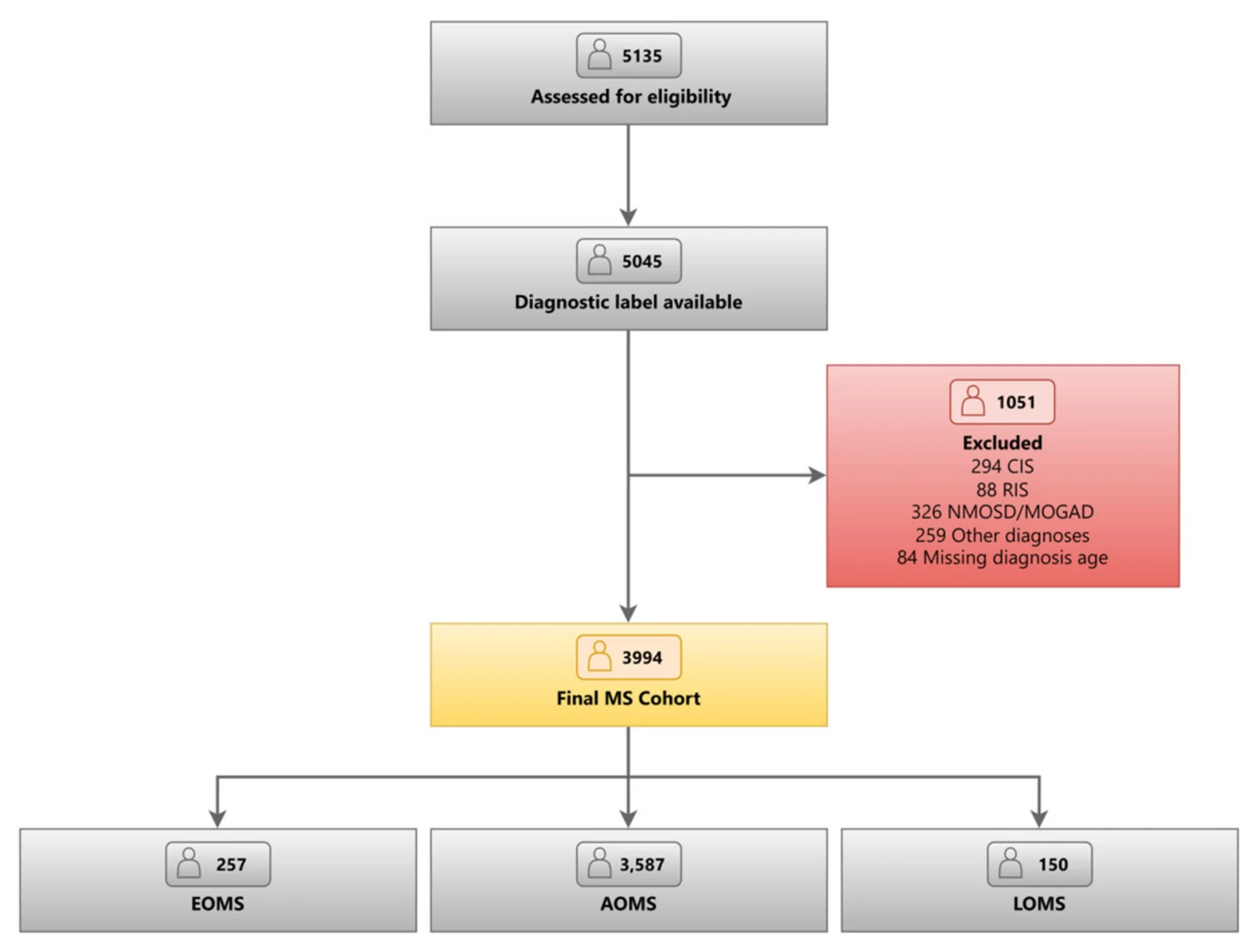
Flow diagram of participant selection process.

Of 5135 patients, the exact diagnostic label at the time of last follow-up was available for 5045 patients. We excluded 294 patients with CIS, 88 patients with RIS, 326 patients with NMOSD/MOGAD, 259 cases with other non-MS diagnoses, and 84 with missing diagnosis age, leaving a total of 3994 patients with MS, comprising 257 patients with EOMS (6.4%), 3587 with AOMS (89.8%), and 150 with LOMS (3.8%), as the final sample for the current study (Figure 1). In terms of disease phenotype, 3186 patients had RRMS (79.8%), 601 had secondary progressive MS (SPMS; 15.0%), and 207 had primary progressive MS (PPMS; 5.2%).

Baseline demographic characteristics differed substantially across the three groups. Mean age increased from 25.21 ± 7.72 years in EOMS to 37.29 ± 8.72 years in AOMS and 58.04 ± 5.14 years in LOMS (*p* < 0.001). Age at diagnosis followed the expected gradient across study groups, with median [interquartile range; IQR] ages of 17 [15–18] years, 30 [25–36] years, and 53.5 [51–57] years in the EOMS, AOMS, and LOMS groups, respectively (*p* < 0.001). Disease duration was longest among patients with EOMS and shortest among those with LOMS, with median durations of 7.7 [2.8–14.4], 5.0 [1.0–9.6], and 1.6 [0.3–5.4] years, respectively (*p* < 0.001). Female patients predominated in all groups, but the proportion of women was highest in EOMS and lowest in LOMS: 81.7% in EOMS, 77.2% in AOMS, and 70.0% in LOMS (*p* = 0.025) (Table 1).

Expectedly, the burden of comorbidities was higher in older patients in the LOMS group. Hyperlipidemia was present in 24.7% of LOMS patients compared with 4.8% of AOMS and 2.3% of EOMS patients (*p* < 0.001). Diabetes mellitus was reported in 18.7% of LOMS, 2.5% of AOMS, and 0.8% of EOMS patients (*p* < 0.001), while hypertension was present in 24.0%, 4.3%, and 0.8%, respectively (*p* < 0.001). Hypothyroidism was also more common in LOMS (18.0%) than in AOMS (10.6%) or EOMS (4.3%) (*p* < 0.001). Hyperthyroidism prevalence was marginally different across groups (*p* = 0.049) (Table 1).

### 3.1. MS Presentation and Phenotypes

MS phenotype differed significantly across the groups (*p* < 0.001). RRMS was the predominant phenotype in all groups but was less frequent among patients with LOMS (Table 2).

RRMS accounted for 85.2% of EOMS, 80.5% of AOMS, and 52.7% of LOMS cases. SPMS was observed in 14.0%, 15.0%, and 18.0% of EOMS, AOMS, and LOMS patients, respectively. PPMS was markedly more common in LOMS, affecting 29.3% of LOMS patients compared with 4.5% of AOMS and 0.8% of EOMS patients. Current DMT class also differed across groups (*p* = 0.016). Injectable therapies were most frequent in AOMS (45.9%) compared with EOMS (36.9%) and LOMS (38.7%), whereas oral therapies were proportionally most frequent in LOMS (35.0% vs. 29.7% in EOMS and 27.0% in AOMS). Monoclonal antibody use was highest in EOMS (33.5%), followed by AOMS (27.1%) and LOMS (26.3%). Treatment patterns also varied substantially according to MS phenotype; monoclonal antibody use was more frequent in several progressive subgroups, whereas treatment distributions among patients with RRMS showed a different pattern (Supplementary Table S1).

**Figure 2:**
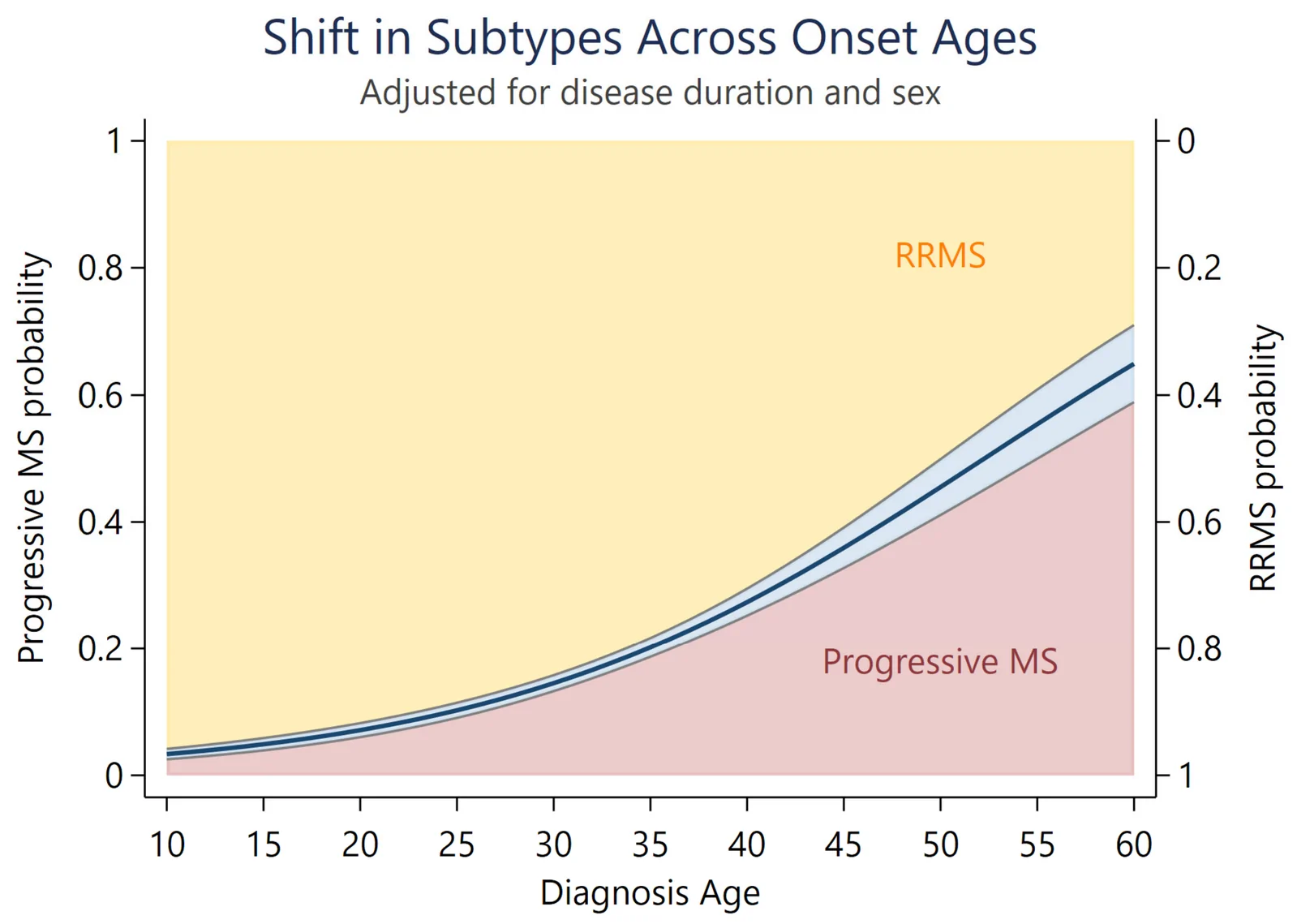
Estimated proportion of patients at study endpoint with progressive MS given the age at diagnosis. The plotted values represent predicted probabilities for an individual, with the mean values of the adjustment variables at each specified diagnosis age rather than crude values observed within age groups. Blue shaded area represents the 95% CI of the estimate line and was calculated using the delta method.

Disability at diagnosis increased across the groups. Median EDSS at diagnosis was 1 [1–2] in EOMS, 2 [1.5–2.5] in AOMS, and 2.5 [2–3.5] in LOMS (*p* < 0.001). There was a positive relationship between the risk of progressive MS vs. RRMS status at mean follow-up (6.4 years) and increasing age at diagnosis, with the estimated proportion of progressive MS patients increasing from 6.1% in patients diagnosed at 18 years of age to 45.5% in those diagnosed at age of 50 (Figure 2).

The shift was mainly due to increasing cases diagnosed with PPMS compared to SPMS, with the former rising from less than 1% of total MS cases in EOMS to 29.3% in rough prevalence among the study groups, while the latter remained relatively stable (14 to 18% within the same period). As there was a significant difference in disease duration between the study groups and in its confounding association with SPMS prevalence, the lack of change may be the result of differences in disease duration and follow-up rather than reflecting a biological age effect. Noticeably, complementary analyses revealed that, while there was a crude association between female preponderance and younger age at diagnosis (OR for female vs. male sex per 1 year increase in diagnosis age: 0.98, 95% CI 0.98–0.99, *p* < 0.001), the term failed to retain significance after adjustment with MS subtypes and to be dependent on and in line with the subtype shift across diagnosis ages (OR: 0.99, 95% CI 0.98–1.00, *p* = 0.072).

MRI characteristics differed across the groups. Brain atrophy was more frequent and more severe among patients with LOMS (*p* < 0.001). No brain atrophy was reported in 77.8% of EOMS, 74.3% of AOMS, and 53.3% of LOMS patients, while moderate or severe atrophy was present in 5.4%, 8.7%, and 15.3%, respectively. A high lesion load was observed in 40.2% of EOMS patients, compared with 32.2% of AOMS and 31.3% of LOMS patients. MRI findings also showed greater chronic disease burden in progressive phenotypes, but active inflammatory MRI lesions were generally more frequent in RRMS than in progressive phenotypes (Supplementary Table S1). In AOMS, no brain atrophy was reported in 84.7% of RRMS patients compared with 27.1% of SPMS and 45.3% of PPMS patients. Moderate or severe brain atrophy was substantially more frequent in SPMS and PPMS than in RRMS. Subsequent adjusted analyses supported the descriptive results, with EOMS (proportional odds ratio; pOR: 0.63, *p* = 0.010) and LOMS (pOR: 1.98, *p* < 0.001) showing lower and higher levels of brain atrophy compared to AOMS, respectively (Table 3).

High lesion load was also more common in SPMS than RRMS across the groups. When stratified by both study group and MS phenotype, patients with SPMS generally had longer disease duration, higher disability at diagnosis, greater MRI burden, and more frequent brain atrophy than patients with RRMS within the same group (Supplementary Table S1).

EOMS had the highest prevalence of enhancing brain lesions in descriptive analyses (*p* = 0.017). The difference between EOMS and AOMS (OR: 1.42, *p* = 0.038) remained significant after adjustment for disease duration and MS subtype (Table 3). Among patients with at least one enhancing brain lesion, lesion counts differed across groups (*p* = 0.016), though estimates in LOMS were based on only 14 patients and should be interpreted cautiously. The presence of active cervical spinal cord plaques did not differ significantly across groups (*p* = 0.491), though the number of active cervical plaques among patients with active lesions differed across groups (*p* < 0.001). Unlike enhancing brain lesions, among patients with at least one cervical plaque regardless of activity, active cervical plaque counts were only higher in EOMS.

### 3.2. Time to First Relapse After Diagnosis

**Figure 3:**
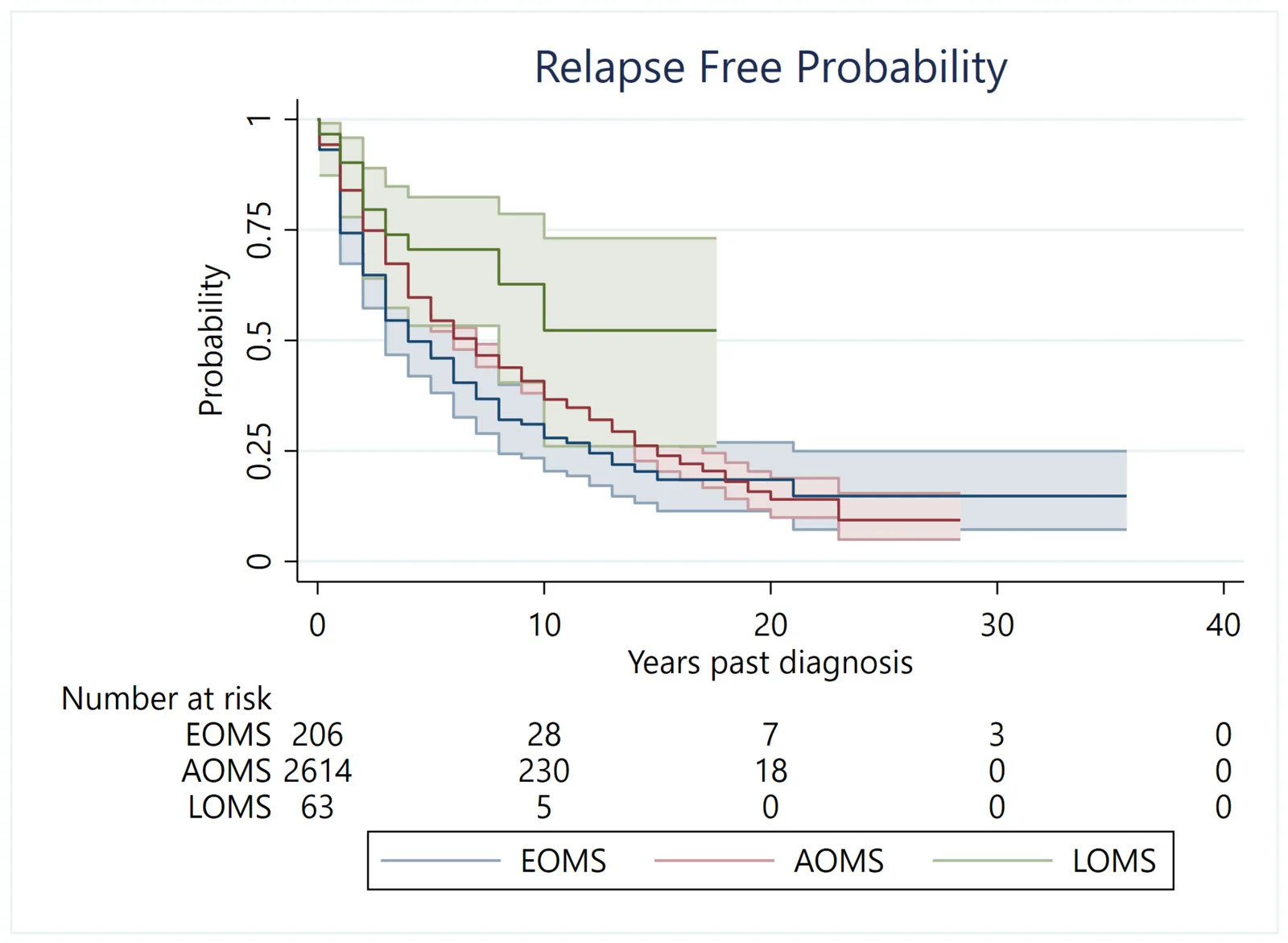
Kaplan–Meier plot for relapse-free probability given the follow-up duration after diagnosis. Patients are derived from a subset of RRMS patients with relapse data available.

Data on relapse follow-up was available for 2883 RRMS patients and were included in the relapse outcome, with 1198 cases with relapses and 11,480 person-years of observation. Patients with progressive MS (PPMS and SPMS) were excluded from relapse-time analyses because relapse-based outcomes are not directly comparable in progressive MS (Figure 3).

The median [IQR] follow-up duration for patients not reaching the outcome (patients without relapse) was 2.70 [0.66–6.43] years. The median time to first post-diagnosis relapse was 2 [1–5] years. There were 120, 1064, and 14 relapse cases in EOMS, AOMS, and LOMS, respectively. In the unadjusted Cox analysis, EOMS was associated with a higher hazard of first post-diagnosis relapse compared with AOMS (HR 1.26, 95% CI 1.04–1.53). In contrast, the association between LOMS and slower first relapse interval was only marginally significant (HR 0.61, 95% CI 0.36–1.03; *p* = 0.062) and only reached statistical significance after adjustment for sex and EDSS at diagnosis (HR 0.58, 95% CI 0.34–0.99; *p* = 0.046) (Table 3).

**Figure 4:**
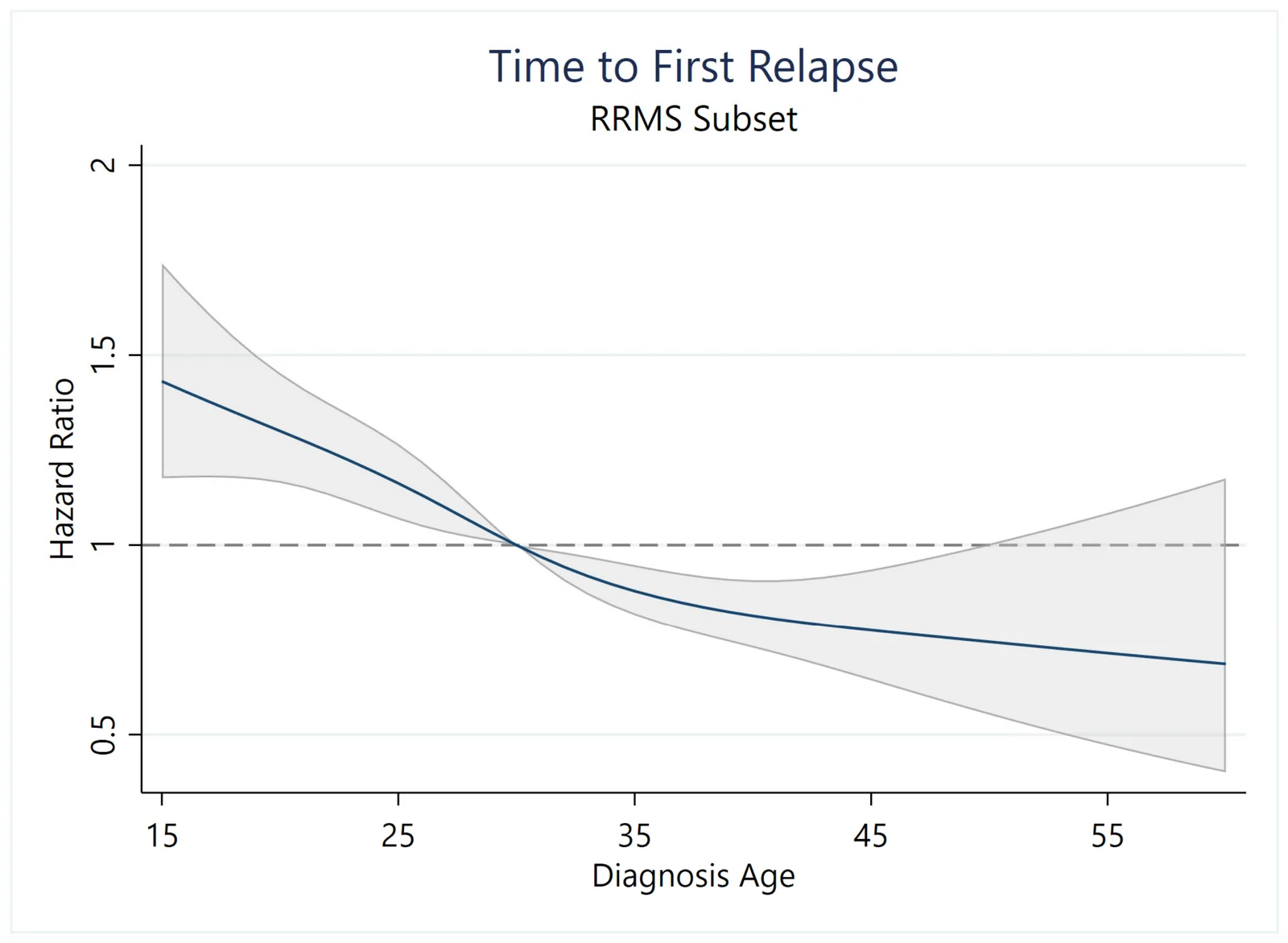
Adjusted estimate of relapse hazard ratio compared to the diagnosis age of 30.

Consistent with the categorical diagnosis age analyses, evaluation of age at diagnosis as a continuous variable showed an inverse association with relapse risk, indicating that increasing age at diagnosis throughout the diagnosis age spectrum was associated with a lower hazard of post-diagnosis relapse (Figure 4).

## 4. Discussion

In this large study of 3994 patients with MS, age at diagnosis was strongly associated with disease phenotype, radiological characteristics, and relapse behavior. EOMS was characterized by a higher hazard of first post-diagnosis relapse and a higher prevalence of active brain plaques, whereas LOMS was characterized by a more progressive and disability-heavy phenotype, including higher EDSS at diagnosis, greater frequency of PPMS, more frequent brain atrophy, higher burden of age-related comorbidities, and a lower hazard of first post-diagnosis relapse. Analyses treating age at diagnosis as a continuous variable demonstrated a progressive increase in the probability of progressive MS and a corresponding reduction in relapse risk across the age spectrum. Collectively, these findings support the view that EOMS, AOMS, and LOMS reflect a continuous age-related shift in disease expression rather than fully discrete clinical entities. This interpretation is consistent with prior work showing that age influences the timing of progression and that the progressive phase of MS is an age-dependent clinical milestone [13,14].

The observed transition from a predominantly relapsing phenotype in younger patients toward a more progressive phenotype in older individuals is also consistent with previous studies of age-related heterogeneity in MS [8,15,16,17]. However, our findings extend prior observations by demonstrating this relationship across multiple clinical and radiological domains within a single large cohort. Importantly, the relationship was not restricted to the comparison of predefined onset groups but was also evident in the analysis across the full age spectrum. This observation is clinically relevant because disability accumulation in MS can occur through mechanisms that are independent of relapse activity, and relapse-independent worsening has been shown to contribute substantially even during the relapsing phase of the disease [18,19]. Accordingly, the higher relapse frequency observed in younger patients may not necessarily translate into faster disability progression [15]. Conversely, the lower relapse hazard observed in LOMS should not be interpreted as evidence of milder disease but rather as a shift toward forms of disease activity that are less visible to conventional relapse-based outcomes [20,21]. This interpretation is further supported by studies demonstrating that disability accumulation in MS is more strongly related to attained age than to age at disease onset itself [6]. Notably, historical studies comparing AOMS and LOMS have reported comparable rates of disability progression despite greater disability levels among older-onset patients, suggesting that age may exert an important influence on disease expression independent of relapse activity [22].

Age-related biological processes may partially contribute to the patterns observed across the age-at-diagnosis spectrum. Aging is characterized by low-grade chronic systemic inflammation, arising from interactions among intrinsic cellular aging, genetic and epigenetic factors, environmental exposures, and persistent infections [23]. Chronic herpesvirus infections, particularly cytomegalovirus (CMV), may further contribute to immune remodeling and inflammatory changes with aging, while Epstein–Barr virus (EBV) has also been implicated in chronic immune stimulation and immune exhaustion [23]. Brain tissue loss in patients with MS also appears to be more pronounced than in healthy aging, possibly reflecting increased oxidative stress, neuronal iron accumulation, enhanced apoptosis, and impaired remyelination [24]. In parallel, immunosenescence is characterized by thymic involution, contraction and functional alteration of the naïve T-cell compartment, expansion of highly differentiated memory and effector T-cell populations, and alterations in regulatory T-cell homeostasis [25]. An inverted CD4+/CD8+ ratio occurs in a subset of older individuals and appears particularly associated with persistent CMV infection [26]. Age-related intrinsic changes in OPCs additionally reduce their ability to differentiate into mature myelinating oligodendrocytes, further limiting remyelination [27,28] and the newer evidence hinting toward axonal transport deficits detectable before the occurrence of neurodegeneration, with impaired transport proposed to contribute to axonal loss through disrupted mitochondrial trafficking and energy homeostasis [29,30]. These mechanisms were not directly assessed in our cohort and therefore should be regarded as possible biological explanations based on the previous literature rather than mechanisms demonstrated by the present findings.

DMT prescribing patterns also differed across the groups in our cohort, though these differences should be interpreted in the context of the marked variation in disease phenotype across age groups. Physicians adopt highly effective therapies in patients with MS starting at lower ages and in patients with more debilitating disease. Thus, the observed treatment distribution likely reflects, at least in part, differences in disease phenotype, severity, inflammatory activity, comorbidity, and treatment selection rather than age at onset alone. Importantly, the greater progressive disease burden observed in LOMS should not be interpreted as indicating an absence of treatment-responsive inflammatory activity. Recent multinational registry data demonstrate that LOMS is associated with greater disability progression and progression independent of relapse activity compared with AOMS [31]. Conversely, another MSBase comparative-effectiveness study in LOMS found that moderate- to high-efficacy DMTs were associated with lower annualized relapse rates and a lower hazard of first relapse than low-efficacy therapies, despite overall low relapse activity [32]. Most confirmed disability progression in the latter study was attributable to progression independent of relapse activity, for which a significant treatment difference was not demonstrated. Thus, the therapeutic implication of aging in MS is that the benefits provided by anti-inflammatory DMTs are confounded by the increasingly prominent relapse-independent component of disability progression.

The attenuation of female predominance with increasing age at diagnosis is also consistent with the prior literature, which suggests that sex hormones and menarche contribute to disease susceptibility [33,34]. Proposed mechanisms include estrogen-related neuroendocrine changes during puberty, such as thymic involution, enhanced CD4+ T-cell survival, and elevated leptin levels during the peri-pubertal period in girls [33,35,36]. However, our findings suggest that age-related changes in sex distribution may also reflect shifts in disease phenotype. Specifically, the increasing prevalence of progressive disease with advancing age may contribute to a more balanced sex ratio [6], as PPMS has consistently demonstrated a substantially lower female predominance than RRMS, with several studies reporting near-equal sex distributions between males and females [37].

The age at diagnosis observed in our EOMS group was also comparable to previous regional reports, including studies from Isfahan [38,39,40] and the United Arab Emirates [41], and the AOMS and LOMS ages were within the ranges reported in prior cohorts [42,43,44]. The higher burden of comorbidities in LOMS is consistent with previous studies showing that older individuals with MS have more vascular, metabolic, and other chronic comorbid conditions, which may complicate diagnosis and contribute to disability, aligning with previous studies on MS patients aged ≥ 45 years [45,46]. While our results are in line with our previous investigations [8], other studies have failed to show significantly different hazards of first relapses between the groups [16].

MRI findings reinforced the same overall pattern. LOMS was associated with more frequent and more severe brain atrophy, which is consistent with the broader literature showing that atrophy is a major marker of irreversible tissue injury and is more closely related to progression than to acute inflammatory activity [47]. By contrast, EOMS showed a greater burden of active brain plaques and more active cervical spinal cord plaques, which is consistent with the more inflammatory phenotype of younger-onset disease [48]. Prior pediatric MS studies describe a more active radiological profile in younger patients, while late-onset MS has been associated with a substantial spinal cord lesion burden and more progressive imaging features [49]. Taken together, these findings suggest that radiological expression also shifts with age, from greater inflammatory activity in younger patients to greater structural injury and cerebral atrophy in older patients, partly driven by cumulative effects of iron dysregulation, mitochondrial dysfunction, and glutamate-mediated excitotoxicity [47].

Several limitations should be considered. Disease duration differed substantially across the study groups. Although disease duration was included in the adjusted analyses and unequal observation time was incorporated into the relapse analysis through individual event and censoring times, age at diagnosis, attained age, and disease duration are closely related and could not be fully separated in this study, especially regarding the brain atrophy analyses. Moreover, the significance of relapse analysis for LOMS patients was only marginally significant, which precludes definitive conclusions on lower relapse rates in LOMS patients compared to patients with AOMS. Also, MRI findings were obtained from routine 1.5-T scans, and brain atrophy and lesion load were visually classified rather than quantified using standardized volumetric methods, which may have introduced measurement variability. SPMS status was also determined at last follow-up. Disease duration differed greatly among groups, and patients with shorter follow-up may simply have had less opportunity to convert from RRMS to SPMS. Finally, MS subtype was determined at the last follow-up; therefore, restricting the relapse analysis to patients classified as RRMS at that time may have excluded patients who transitioned to SPMS during follow-up.

## 5. Conclusions

Our results show that age at clinical MS diagnosis is associated with differences in phenotype, MRI findings, comorbidity burden, and relapse outcomes in this observational cohort. Younger diagnosis age was associated with more enhancing lesions and earlier relapse, whereas older diagnosis age was associated with more PPMS, higher disability at diagnosis, and more visually reported atrophy. These findings identify age-associated patterns that require confirmation in prospective cohorts with standardized MRI, complete treatment histories, and reliable symptom-onset dates.

## Figures and Tables

**Table 1:** Demographic characteristics and comorbidities of all patients included in the analyses.

Variable	Number Available	EOMS 257 (6.4%)	AOMS 3587 (89.8%)	LOMS 150 (3.8%)	Total 3994 (100.0%)	Significance
**Age at last visit (mean, SD)**	3994	25.21 (7.72)	37.29 (8.72)	58.04 (5.14)	37.30 (9.93)	<0.001
**Sex (group-wise frequency percentage)**						
Male	3994	47 (18.3%)	819 (22.8%)	45 (30.0%)	911 (22.8%)	0.025
Female		210 (81.7%)	2768 (77.2%)	105 (70.0%)	3083 (77.2%)	
**BMI**	3975	22.50 (3.82)	24.47 (4.31)	26.45 (4.508)	24.42 (4.33)	<0.001
**Marital status**						
Single	3835	172 (68.8%)	752 (21.8%)	8 (5.7%)	932 (24.3%)	<0.001
Married		78 (31.2%)	2693 (78.2%)	132 (94.3%)	2903 (75.7%)	
**Education level**						
Low education	3909	40 (16.0%)	603 (17.2%)	77 (53.1%)	720 (18.4%)	<0.001
Secondary education		106 (42.4%)	1274 (36.2%)	46 (31.7%)	1426 (36.5%)	
Higher education		104 (41.6%)	1637 (46.6%)	22 (15.2%)	1763 (45.1%)	
**Occupation**						
Unemployed/retired	3911	103 (41.4%)	2134 (60.7%)	121 (83.4%)	2358 (60.3%)	<0.001
Employed		41 (16.5%)	1208 (34.3%)	24 (16.5%)	1273 (32.5%)	
Student		105 (42.2%)	175 (5.0%)	0 (0%)	280 (7.2%)	
**Smoking**	3994	13 (5.1%)	333 (9.3%)	18 (12.0%)	364 (9.1%)	0.034
**Headache**	3994	47 (18.3%)	798 (22.2%)	33 (22.0%)	878 (22.0%)	0.334
**Migraine**	3994	20 (7.8%)	407 (11.3%)	14 (9.3%)	441 (11.0%)	0.168
**Hyperlipidemia**	3994	6 (2.3%)	171 (4.8%)	37 (24.7%)	214 (5.4%)	<0.001
**Diabetes mellitus**	3994	2 (0.8%)	90 (2.5%)	28 (18.7%)	120 (3.0%)	<0.001
**Hypertension**	3994	2 (0.8%)	156 (4.3%)	36 (24.0%)	194 (4.9%)	<0.001
**Anemia**	3994	23 (8.9%)	385 (10.7%)	19 (12.7%)	427 (10.7%)	0.488
**Hyperthyroidism**	3994	0 (0.0%)	42 (1.2%)	4 (2.7%)	46 (1.2%)	0.049
**Hypothyroidism**	3994	11 (4.3%)	379 (10.6%)	27 (18.0%)	417 (10.4%)	<0.001
**Seizure**	3994	14 (5.4%)	114 (3.2%)	6 (4.0%)	134 (3.4%)	0.135
**Rheumatologic diseases**	3994	1 (0.4%)	44 (1.2%)	4 (2.7%)	49 (1.2%)	0.132

**Table 2:** Clinical determinants and MS phenotype among study groups.

Variable	Number Available	EOMS 257 (6.4%)	AOMS 3587 (89.8%)	LOMS 150 (3.8%)	Total 3994 (100.0%)	Significance
**Age at diagnosis (mean, SD)**	3994	15.87 (2.43)	30.81 (7.44)	54.53 (4.07)	30.74 (9.28)	<0.001
Diagnosis year (median, IQR) *	3994	2011 [2005–2015]	2013 [2009–2018]	2017 [2013–2020]	2013 [2009–2018]	<0.001
**Disease duration (median, IQR)**	3994	7.7 [2.8–14.4]	5 [1–9.6]	1.6 [0.3–5.4]	5 [1–9.8]	<0.001
**MS phenotype**						
Relapsing–remitting MS	3994	219 (85.2%)	2888 (80.5%)	79 (52.7%)	3186 (79.8%)	<0.001
Secondary progressive MS		36 (14.0%)	538 (15.0%)	27 (18.0%)	601 (15.0%)	
Primary progressive MS		2 (0.8%)	161 (4.5%)	44 (29.3%)	207 (5.2%)	
**EDSS at diagnosis**	3822	1 [1–2]	2 [1.5–2.5]	2.5 [2–3.5]	2 [1.5–2.5]	<0.001
**Current drug type**						
Injection (interferon beta, glatiramer acetate)	3720	87 (36.9%)	1537 (45.9%)	53 (38.7%)	1677 (45.1%)	0.016
Monoclonal antibody (rituximab, natalizumab)		79 (33.5%)	906 (27.1%)	36 (26.3%)	1021 (27.4%)	
Oral (fingolimod, dimethyl fumarate, teriflunomide, mycophenolate mofetil)		70 (29.7%)	904 (27.0%)	48 (35.0%)	1022 (27.5%)	
**Brain atrophy**						
No atrophy	3994	200 (77.8%)	2664 (74.3%)	80 (53.3%)	2944 (73.7%)	<0.001
Mild		43 (16.7%)	613 (17.1%)	47 (31.3%)	703 (17.6%)	
Moderate		9 (3.5%)	254 (7.1%)	20 (13.3%)	283 (7.1%)	
Severe		5 (1.9%)	56 (1.6%)	3 (2.0%)	64 (1.6%)	
**Brain lesion load**						
Low	3500	39 (17.8%)	880 (27.9%)	33 (25.2%)	952 (27.2%)	0.013
Medium		92 (42.0%)	1257 (39.9%)	57 (43.5%)	1406 (40.2%)	
High		88 (40.2%)	1013 (32.2%)	41 (31.3%)	1142 (32.6%)	
**Active brain plaque (vs. no active plaques)**	3994	48 (18.7%)	479 (13.3%)	14 (9.3%)	541 (13.5%)	0.017
**Active brain plaques (count, median IQR) ^#^**	3994	2 [1–5]	1 [1–3]	2.5 [1–5]	1 [1–3]	0.016
**Active cervical spine plaque (vs. no active plaques)**	3994	12 (4.7%)	171 (4.8%)	4 (2.7%)	187 (4.7%)	0.491
**Active cervical spine plaques (count) ^#^**	3994	2 [1–3]	1 [1–1]	1 [1–1]	1 [1–1]	<0.001

* Approximate conversion to Gregorian calendar year; ^#^ lesion count was calculated among patients with at least one active lesion.

**Table 3:** Association of MS onset category with time to first relapse, brain atrophy and active brain plaques (enhancing brain lesions) in regression analyses.

Terms	Univariate Model	Adjusted Model
**Time to first relapse after diagnosis (Cox proportional hazards) (*n* = 2883/2789)**		
**MS onset category (vs. AOMS)**	Ref. (hazard ratio [95% CI], *p*-value)	Ref. (hazard ratio [95% CI], *p*-value)
EOMS	1.26 [1.04–1.53], *p* = 0.016	1.27 [1.05–1.54], *p* = 0.013
LOMS	0.61 [0.36–1.03], *p* = 0.062	0.58 [0.34–0.99], *p* = 0.046
**Sex (female vs. male)**	-	0.93 [0.80–1.08], *p* = 0.360
**EDSS at diagnosis (per unit)**	-	1.12 [1.06–1.18], *p* < 0.001
**Brain atrophy levels (ordinal regression) (*n* = 3994/3822)**		
**MS onset category (vs. AOMS)**	Ref. (proportional odds ratio [95% CI], *p*-value)	Ref. (proportional odds ratio [95% CI], *p*-value)
EOMS	0.81 [0.60–1.09], *p* = 0.163	0.63 [0.44–0.89], *p* = 0.010
LOMS	2.36 [1.73–3.22], *p* < 0.001	1.98 [1.36–2.89], *p* < 0.001
**Sex**	-	0.92 [0.76–1.11], *p* = 0.386
**EDSS at diagnosis**	-	1.19 [1.11–1.27], *p* < 0.001
**Disease duration (per year)**	-	1.09 [1.08–1.11], *p* < 0.001
**MS subtype (vs. RRMS)**	-	Ref.
SPMS	-	7.66 [6.11–9.61], *p* < 0.001
PPMS	-	5.17 [3.71–7.19], *p* < 0.001
**Hypertension**	-	1.11 [0.77–1.59], *p* = 0.576
**Diabetes mellitus**	-	1.09 [0.71–1.68], *p* = 0.698
**Active brain plaque presence (logistic regression) (*n* = 3994/3994)**		
**MS onset category (vs. AOMS)**	Ref. (odds ratio [95% CI], *p*-value)	Ref. (odds ratio [95% CI], *p*-value)
EOMS	1.49 [1.07–2.07], *p* = 0.017	1.42 [1.02–1.99], *p* = 0.038
LOMS	0.67 [0.38–1.17], *p* = 0.157	0.87 [0.49–1.54], *p* = 0.638
**Disease duration**	-	1.00 [0.99–1.02], *p* = 0.663
**MS subtype (vs. RRMS)**	-	Ref.
SPMS	-	0.43 [0.30–0.60], *p* < 0.001
PPMS	-	0.29 [0.15–0.56], *p* < 0.001

## Data Availability

The dataset used to reach the conclusions of this work is available from the corresponding author on reasonable request.

## Supplementary Materials

The following supporting information can be downloaded at https://www.mdpi.com/article/10.3390/neurosci7050111/s1, Table S1: Clinical determinants and MS phenotype of included patients stratified by onset groups.

## Abbreviations

The following abbreviations are used in this manuscript:

MS	Multiple sclerosis
CNS	Central nervous system
AOMS	Adult-onset MS
POMS	Pediatric-onset MS
EOMS	Early-onset MS
LOMS	Late-onset MS
CIS	Clinically isolated syndrome
RIS	Radiologically isolated syndrome
RRMS	Relapsing–remitting MS
SPMS	Secondary progressive MS
PPMS	Primary progressive MS
OR	Odds ratio
HR	Hazard ratio
CD	Cluster of differentiation
