# Supplementary material for: Association of Diagnosis Age with Clinical Characteristics and MRI Findings in Multiple Sclerosis
Source: NeuroSci. 2026 Oct 9;7(5):111. doi: 10.3390/neurosci7050111 (PMC13708649; doi:10.3390/neurosci7050111)
Supplement: Supplementary file 1 [file neurosci-07-00111-s001.zip › neurosci-4573874-supplementary.pdf]

Supplementary Table S1: Clinical determinants and MS phenotype of included patients stratified by onset groups.

Disease characteristics	EOMS			AOMS			LOMS		
	RRMS (n=219)	SPMS (n=34)	PPMS* (n=2)	RRMS (n=2,888)	SPMS (n=538)	PPMS (n=161)	RRMS (n=79)	SPMS (n=27)	PPMS (n=44)
**Age at diagnosis (Mean, SD)**	15.84 (2.52)	16.08 (1.79)	-	30.37 (7.22)	31.59 (7.77)	36.10 (7.87)	53.59 (3.43)	54.41 (3.82)	56.29 (4.74)
**Disease duration**	6.6 [2.5 – 12.4]	15.2 [11.0 – 18.8]	-	4.1 [0.7 – 8.3]	11.9 [7.4 – 17.7]	4.7 [0.5 – 10.3]	1.7 [0.2 – 5.6]	6.8 [2.3 – 10.5]	0.6 [0.1 – 1.9]
**EDSS at diagnosis**	2 [0 – 2]	2 [2 – 3.25]	-	2 [1 – 2]	3 [2 – 4.5]	3 [2.25 – 4.5]	2 [1.5 – 2.5]	3 [2 – 4]	4 [2.75 – 5.5]
**Current drug type**									
Injection	85 (41.9%)	2 (6.5%)	-	1,427 (52.0%)	94 (21.9%)	16 (11.0%)	35 (46.1%)	10 (47.6%)	8 (20.0%)
Monoclonal antibody	52 (25.6%)	25 (80.6%)	-	521 (18.5%)	271 (63.0%)	114 (78.6%)	8 (10.5%)	3 (14.3%)	25 (62.5%)
Oral	66 (32.5%)	4 (12.9%)	-	824 (29.5%)	65 (15.1%)	15 (10.3%)	33 (43.4%)	8 (38.1%)	7 (17.5%)
**Brain atrophy**									
No atrophy	192 (87.7%)	7 (19.4%)	-	2,445 (84.7%)	146 (27.1%)	73 (45.3%)	55 (69.6%)	5 (18.5%)	20 (45.5%)
Mild	23 (10.5%)	20 (55.6%)	-	373 (12.9%)	192 (35.7%)	48 (29.8%)	19 (24.1%)	12 (44.4%)	16 (36.4%)
Moderate	3 (1.4%)	6 (16.7%)	-	63 (2.2%)	155 (28.8%)	36 (22.4%)	4 (5.1%)	9 (33.3%)	7 (15.9%)
Severe	1 (0.5%)	3 (8.3%)	-	7 (0.2%)	45 (8.4%)	4 (2.5%)	1 (1.3%)	1 (3.7%)	1 (2.3%)
**Lesion load**									
Low	38 (20.5%)	0 (0.0%)	-	823 (32.5%)	31 (6.6%)	26 (17.6%)	19 (28.8%)	2 (8.0%)	12 (30.0%)
Medium	88 (47.6%)	4 (12.5%)	-	1,114 (44.0%)	95 (20.2%)	48 (32.4%)	34 (51.5%)	10 (40.0%)	13 (32.5%)
High	59 (31.9%)	28 (87.5%)	-	595 (23.5%)	344 (73.2%)	74 (50.0%)	13 (19.7%)	13 (52.0%)	15 (37.5%)
**Active brain plaque (vs. no active plaques)**	43 (19.6%)	5 (13.9%)	-	433 (15.0%)	39 (7.2%)	7 (4.3%)	11 (13.9%)	0 (0.0%)	3 (6.8%)
**Active brain plaques (count)**	2 [1 – 3]	5 [3 – 10]	-	1 [1 – 3]	2 [1 – 3]	1 [1 – 3]	3 [1 – 5]	0	1 [1 – 3]
**Cervical spine plaque (vs. no active plaques)**	12 (5.5%)	0 (0.0%)	-	148 (5.1%)	16 (3.0%)	7 (4.3%)	2 (2.5%)	1 (3.7%)	1 (2.3%)
**Active Cervical spine plaques (count)**	2 [1 – 3]	0	-	1 [1 – 1]	1 [1 – 2]	1 [1 – 2]	1 (both)	1	1

*Summary results were not reported for the PPMS subset
